# Supplementary material for: Seminoma and Embryonal Carcinoma Footprints Identified by Analysis of Integrated Genome-Wide Epigenetic and Expression Profiles of Germ Cell Cancer Cell Lines
Source: PLoS One. 2014 Jun 2;9(6):e98330. doi: 10.1371/journal.pone.0098330 (PMC4041891; doi:10.1371/journal.pone.0098330)
Supplement: File S1 — ZIP file containing DMRforPairs output for significant regions. Please start from the html files. (ZIP) [file pone.0098330.s008.zip › figures/35906.pdf]

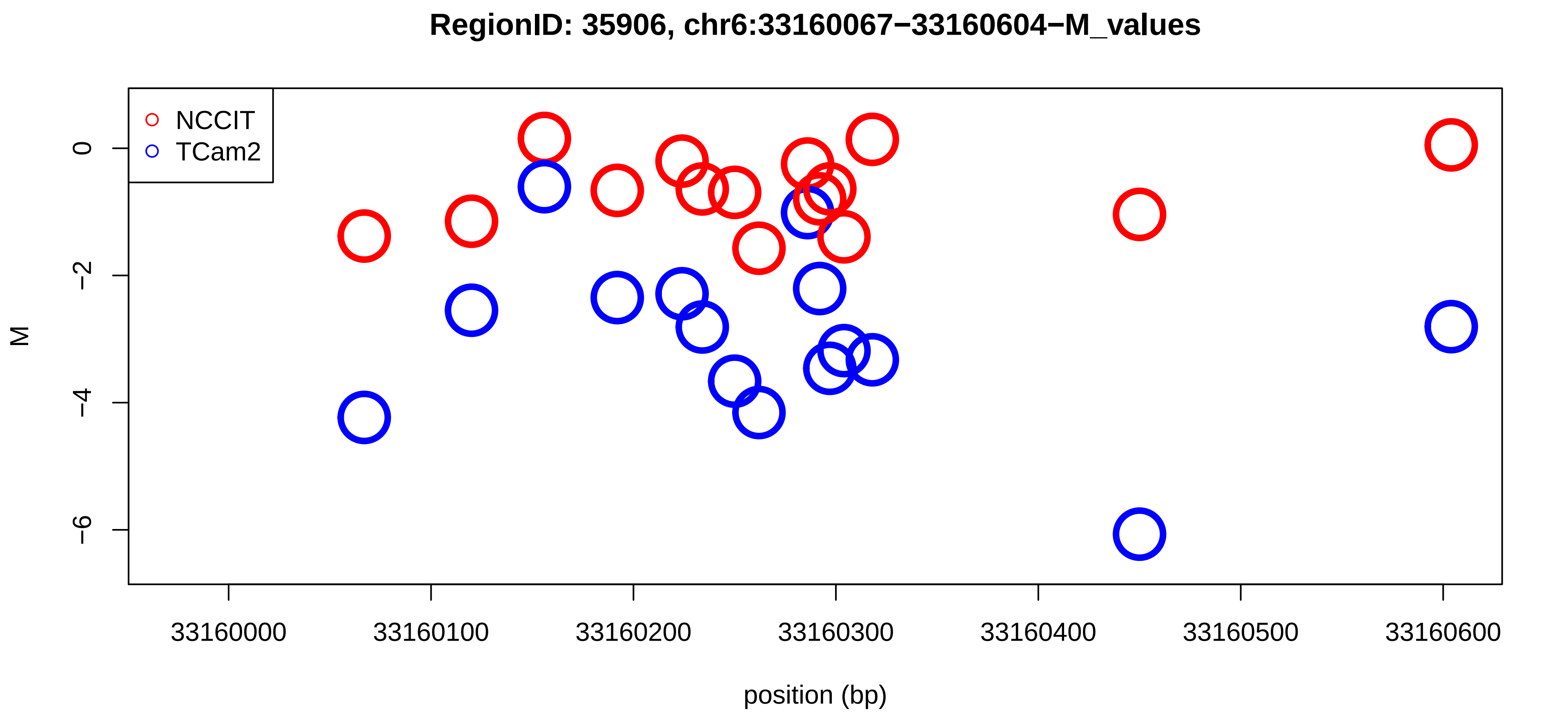

RegionID: 35906, chr6:33160067−33160604−M_values
NCCIT
TCam2
M
0
−2
−4
−6
33160000
33160100
33160200
33160300
33160400
33160500
33160600
position (bp)

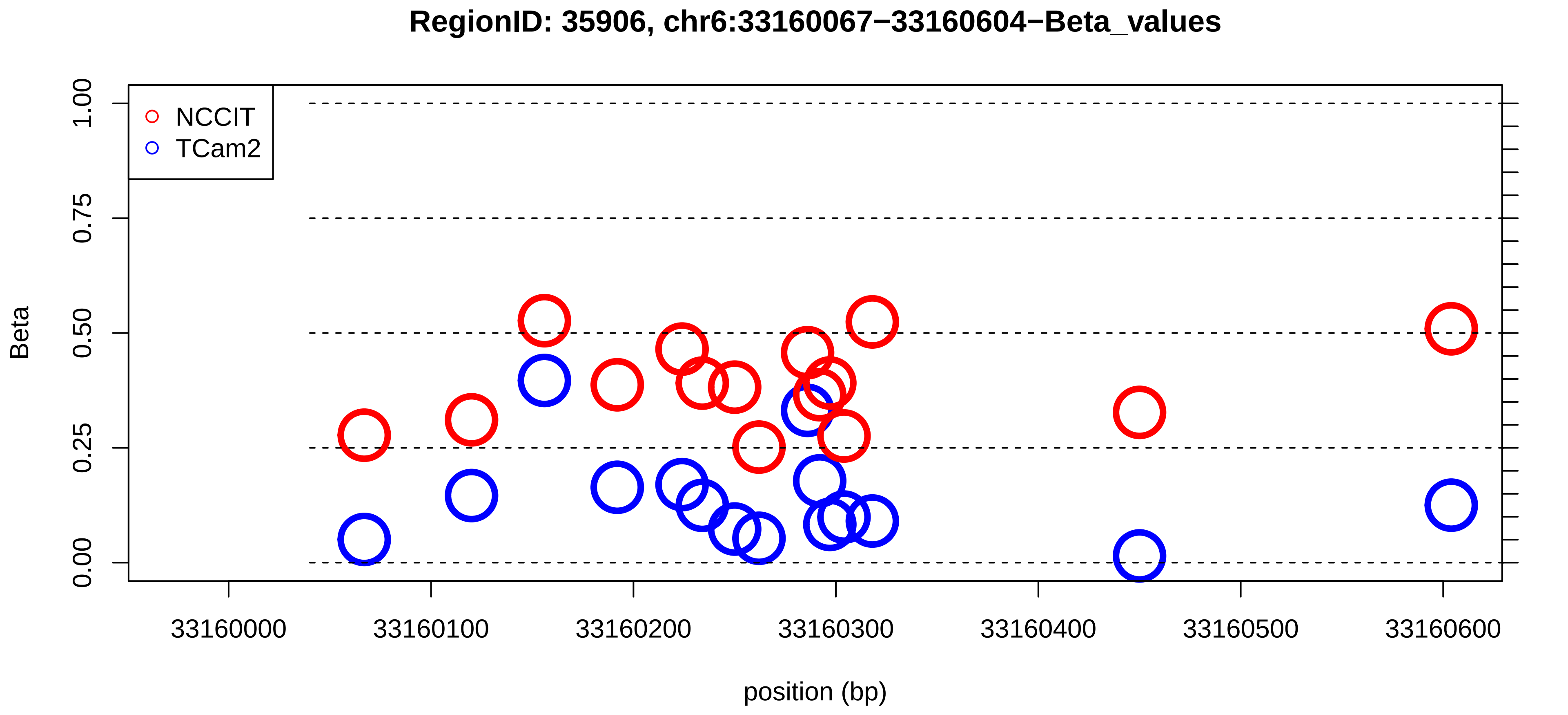

RegionID: 35906, chr6:33160067−33160604−Beta_values
NCCIT
TCam2
Beta
1.00
0.75
0.50
0.25
0.00
33160000
33160100
33160200
33160300
33160400
33160500
33160600
position (bp)

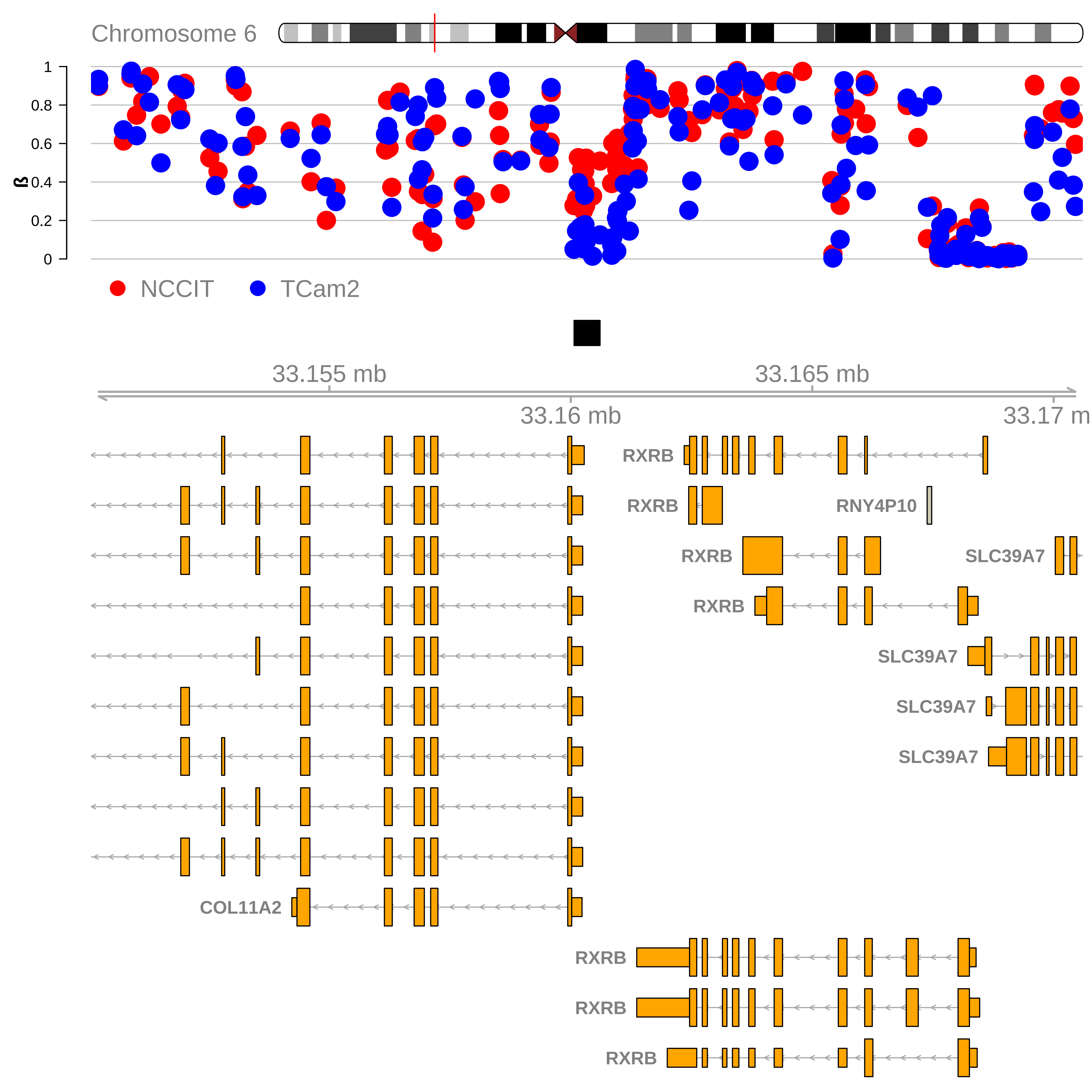

Chromosome 6
1
0.8
0.6
0.4
0.2
0
ß
NCCIT
TCam2
33.155 mb
33.165 mb
33.16 mb
33.17 m
RXRB
RXRB
RNY4P10
RXRB
SLC39A7
RXRB
SLC39A7
SLC39A7
SLC39A7
COL11A2
RXRB
RXRB
RXRB
